# Towards Precision Aging Biology: Single-Cell Multi-Omics and Advanced AI-Driven Strategies

**DOI:** 10.14336/AD.2025.0218

**Published:** 2025-03-13

**Authors:** Sijia Xie, Xinwei Luo, Feitong Hong, Yijie Wei, Yuduo Hao, Xueqin Xie, Xiaolong Li, Guangbo Xie, Fuying Dao, Hao Lyu

**Affiliations:** ^1^The Clinical Hospital of Chengdu Brain Science Institute, School of Life Science and Technology, University of Electronic Science and Technology of China, Chengdu, 610054, China.; ^2^Center for Informational Biology, School of Life Science and Technology, University of Electronic Science and Technology of China, Chengdu, 610054, China.; ^3^School of Biological Sciences, Nanyang Technological University, Singapore 639798, Singapore.

**Keywords:** Single-cell omics, Cell senescence, Artificial Intelligence, Biomarker

## Abstract

Individual aging is a complex biological process involving multiple levels, with molecular changes existing in heterogeneity across different cell types and tissues, being regulated by both internal and external factors. Traditional senescence markers, including p16, cell morphological changes, and cell cycle arrest, can only partially reflect the complexity of senescence. Single-cell omics technology facilitates the integration of multi-faceted data, including gene expression profiles, spatial dynamics, chromatin accessibility and metabolic pathways. This comprehensive approach enhances the development of biomarkers, granting us a more profound insight into the heterogeneity inherent within senescent cell populations. In this review, we summarize the application of single cell multi-omics approaches in analyzing senescence mechanisms and potential intervention targets from the perspectives of transcriptomics, epigenetics, metabolomics, and proteomics, explore the potential of developing new senescence markers at the cellular level using machine learning algorithms and artificial intelligence in bioinformatics analysis. Finally, we further discuss the challenges and prospective trajectories within this research domain to provide a more comprehensive perspective on dissecting the regulatory networks of senescence cells.

## Introduction

The concept of senescent cells was first proposed by the "Hayflick limit" theory, which describes how cells enter a state of persistent non-proliferative after experiencing stress or damage [1]. This physiological process is closely related to multiple molecular mechanisms, including disordered cell cycle regulation, telomere shortening, DNA damage accumulation, mitochondrial function decline, and oxidative stress [2]. Furthermore, senescent cells are not completely static, they release a plethora of chemokines, cytokines, growth factors and proteases, collectively forming the senescence-associated secretory phenotype (SASP), which promotes chronic inflammation in surrounding tissues and accelerates the aging process [3]. In recent years, extensive aging-related research has deepened our understanding of the physiological functions and underlying molecular mechanisms of cellular senescence. Notably, the SenNet Consortium proposed nine core hallmarks of cellular senescence and characterized tissue-specific senescence markers [4].

However, senescence exhibits significant heterogeneity across different cell types and tissues, different rates of cellular aging can be observed among cell types and even between subpopulations [5]. Conventional bulk tissue analysis fails to define the function of specific cell types or tissues based on the expression of one or a few genes, often masking the diverse aging characteristics exhibited by individual cells within a population. This limited perspective hinders our ability to pinpoint the cellular origins of age-related decline and develop effective interventions. The advent of single-cell sequencing technologies-including trans-criptomics, epigenomics, metabolomics, proteomics, and spatial omics, has revolutionized aging research by offering unprecedented resolution. These advancements reveal the complex heterogeneity of senescence states across cell types, as well as their dynamic changes and intricate interactions in space and time [6]. Such insights enable a more systematic analysis of aging and provide new avenues for delaying aging and preventing age-related diseases.

Accurate identification of senescent cell populations and their biomarkers is crucial for devising interventions and addressing age-related diseases. However, current insights into cellular senescence states are often fragmented across studies that emphasize particular omics layers, tissues, pathological conditions, or therapies [7]. Single-cell multi-omics analysis strategies, while promising, face significant challenges in data integration [8]. Fully harnessing computational approaches and artificial intelligence (AI) for the integrative analysis of multidimensional data to identify cellular senescence features is essential for accelerating the discovery of senescence biomarkers and uncovering the mysteries of aging [9].

**Figure 1.**
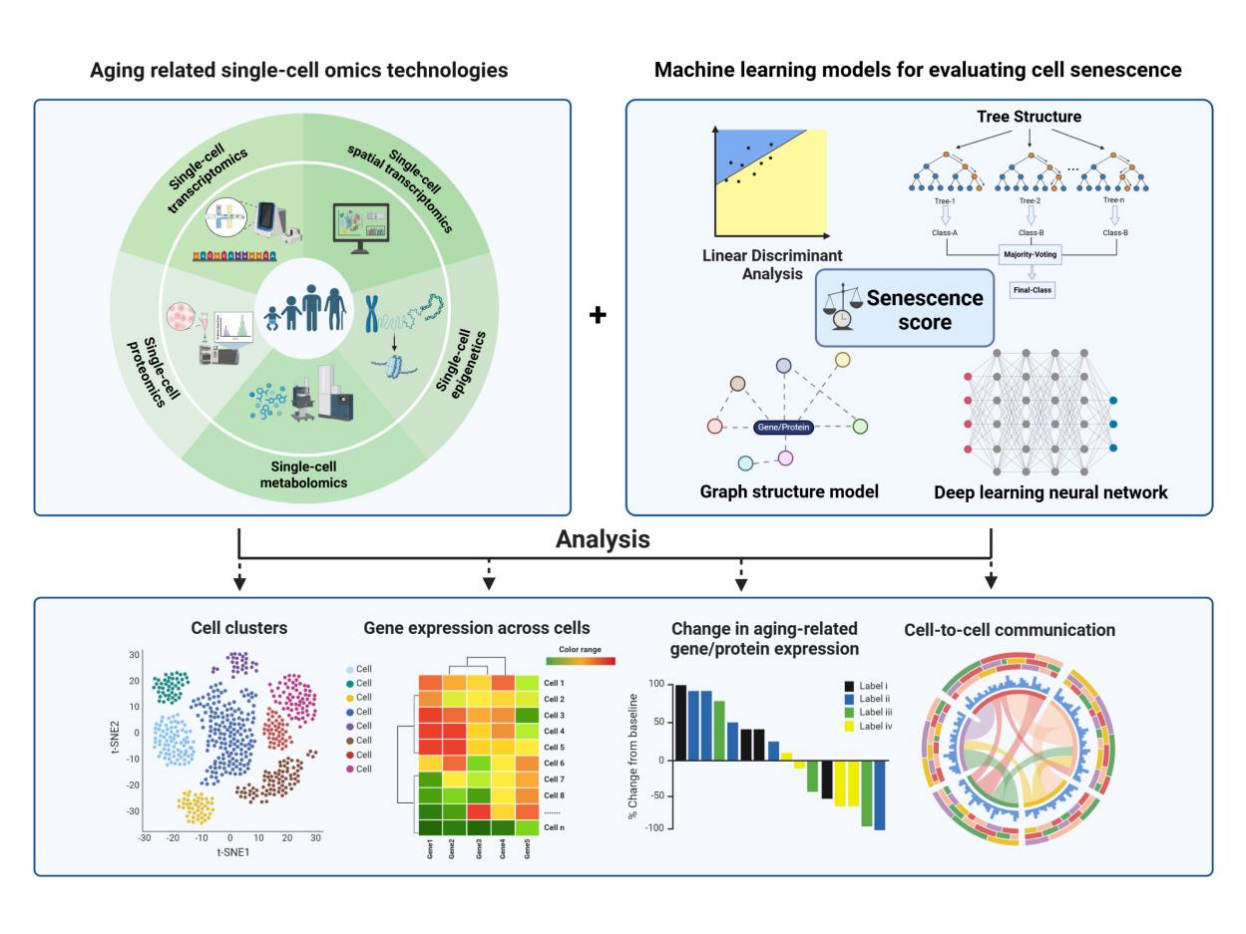
**A framework for the application of single-cell technology combined with machine learning in cellular senescence research**. Single-cell multiomics technology empowers the generation of high-resolution data, while the machine learning model serves as a "decoder". By leveraging this combination, it becomes possible to meticulously dissect the characteristics pertaining to cell types, gene expression patterns, as well as the dynamics of intercellular communication at the single-cell level. Figure created using BioRender.

In this review, we systematically revisit the applications of single-cell omics techniques in identifying senescent cell types and uncovering key features of senescence. Additionally, we explore recent advances in decoding senescence markers at the single-cell level using AI-based methods (Fig. 1). Addressing current research strategies in the field of aging, we critically analyze their limitations and discuss potential solutions. Ultimately, we aim to provide innovative insights for aging research and pave the way for mitigating aging and promoting healthy longevity.

### Advancements in single-cell omics technologies for aging study: unveiling cellular heterogeneity and senescence mechanisms

The study of multidimensional biological information at the single-cell level, including gene expression, metabolic states, and epigenetic modifications, has generated increasing attention. The high-dimensional data generated by these technologies provide valuable resources for investigating gene expression changes during cellular senescence, identifying key regulatory networks, and predicting the dynamics of senescence-related biological processes (Table 1).

**Table 1:** Single-cell omics aging datasets.

Study	Dataset	Single-cell Analyses	Methods	Species	Tissue	Age
**Ximerakis et al. [17]**	GSE129788	Transcriptomics	10x Genomics	Mouse	Brain	Young (2-3 months) Old (21-23 months)
**Green et al. [18]**	syn31512863	Transcriptomics	snRNA-seq	Human	Brain (Prefrontal cortex)	The average age of death was 89.1 years
**Isola et al. [19]**	GSE232309	Transcriptomics	10x Genomics	Mouse	Ovary	Young (3 months) Reproductively aged (9 months)
**Zhou et al. [20]**	/	Transcriptomics	10x Genomics	Human	Ovary	Young (< 33 years) Middle-aged (> 37 years)
**Nie et al. [21]**	GSE182786	Transcriptomics	10x Genomics	Human	Testis	Young (17-22 years) Old (>60 years)
**Zhang et al. [22]**	OMIX1000	Transcriptomics	Drop-Seq	Mouse	Testis	Young (2 months) Old (24 months)
**Wu et al. [30]**	GSE193107	Spatial transcriptomics	Visium Spatial (10X Genomics)	Mouse	Brain	Young (2 months) Old (28 months)
**Allen et al. [31]**	GSE207848 CELL x GENE repository (https://cellxgene.cziscience.com/collections/31937775-0602-4e52-a799-b6acdd2bac2e)	Spatial transcriptomics	10X Genomics MERFISH	Mouse	Frontal cortex and striatum	Juvenile (1 month) Old (21 months)
**Wu et al. [32]**	GSE255690	Spatial transcriptomics	Visium Spatial (10x Genomics)	Human	Ovary	Young (18 - 28 years) Middle-aged (36 - 39 years) Old (47 - 49 years)
**Winkler et al. [33]**	MTAB-11491 MTAB-12889 E-MTAB-12105	Spatial transcriptomics	Visium Spatial (10x Genomics)	Mouse	Female reproductive tract	Young (3 months) Old (18 months)
**Ma et al. [34]**	STT0000039 CRA012965	Spatial transcriptomics	Stereo-seq (10x Genomics)	Mouse	Multiple tissues	Young (2 months) Old (25 months)
**Xiao et al. [45]**	https://github.com/caleblareau/mtscATACpaper_reproducibility	Epigenomics	scATAC-seq Whole-genome bisulfite sequencing Mass cytometry (EpiTOF)	Human Mouse	Multiple tissues	Human (aged 22-70 years) Mouse (aged 7-403 days)
**Trapp et al. [46]**	GSE68642 GSE121436 GSE56879 GSE121690 SRA344045 GSE120132	Epigenomics	Whole genome methylation sequencing	Mouse	1.Liver 2.Skeletal muscle	1.Young (4 months) Old (26 months) 2.Young (1.5 months) Old (26 months)
**Cheung et al. [48]**	/	Epigenomics	Mass cytometry (EpiTOF)	Mouse	PBMCs	Young (< 25 years) Old (> 65 years)
**Alvarez-Kuglen et al. [49]**	https://osf.io/mkc9u/	Epigenomics	Whole genome methylation sequencing	Human	PBMCs	Young (2 months) Old (27 months)
**Liu et al. [55]**	/	Metabolomics	SlipChip-SERS	Mouse	Multiple tissues	Young (9 years) Old (92,87,81years)
**Xu et al. [58]**	AAGMEx cohort	Metabolomics	LC-MS	Human	Adipose and muscle	18-61 years
**Zhang et al. [59]**	UK Biobank	Metabolomics	NMR	Human	Blood	40-70 years
**Huang et al. [61]**	CRA008819 HRA00340 HRA004706	Proteomics	MS	Human	Oocyte	Young (4 weeks), old (>16 months) for mouse Young (≤31 years), old (≥38 years) for human
**Tracey et al. [65]**	/	Proteomics	CyTOF	Mouse and Human	Multiple tissues	/

### Single-cell transcriptomics

Single-cell transcriptomics, as the most mature and widely applied single-cell omics technology, provides high-resolution insights into gene expression and transcriptional regulation under specific conditions [10]. Since the first implementation of single-cell transcriptome sequencing (scRNA-seq) in 2009 [11], the field has witnessed rapidly advancements. It has evolved from sequencing randomly selected individual cells to large-scale, multiplexed sequencing of tens of thousands of cells [12]. This technology has two major branches: microfluidics droplet-based transcriptomics and microwell plate-based transcriptomics sequencing [13]. Although the principles of these two technologies differ, their data processing workflows are similar, These workflows typically include sample preparation and sequencing, alignment of raw reads, preprocessing and normalization of count matrices, cell clustering and annotation, differential expression analysis, dimensionality reduction, visualization, and downstream analysis [14].

scRNA-seq and single-nucleus RNA-sequencing (snRNA-seq) are powerful tools for investigating the heterogeneity of aging [15]. They enable the observation of molecular-level changes in individual cells and the characterization of transcriptomic features. Compared to snRNA-seq, scRNA-seq provides transcriptomic information from both the cytoplasm and nucleus. However, the integrity of the data may be compromised due to damage to fragile senescent cells caused by isolation techniques. In contrast, snRNA-seq causes minimal damage to cells but has lower sequencing sensitivity due to its inability to capture cytoplasmic mRNA.

Despite these challenges, recent advances in ultra-high-throughput scRNA-seq have shown the potential to depict entire organisms with unprecedented resolution. Researchers can reveal the dynamic changes in gene expression in specific cell types during the aging process [16]. This allows them to address key questions, such as: “Which genes are upregulated or downregulated in senescence cells?”, “What are the differences in gene expression between various cell types?”, and “What are the differentiation trajectories, fate transitions, and molecular mechanisms of cells during aging?”. For instance, an scRNA-seq analysis of the mouse brain revealed significant differences in the senescence characteristics of different brain cell types [17]. Notably, expression of ribosomal protein genes showed opposite aging trends among different brain cell subpopulations and neuronal subtypes. While reduced ribosomal protein gene expression and protein synthesis have traditionally been considered hallmarks of aging, some studies have observed increased ribosomal protein gene expression during aging. This paradox may reflect compensatory mechanisms to cope with aging-induced metabolic changes or the production of specific types of ribosomes to meet translational demands. Futhermore, previous studies have demonstrated that the human brain aging is closely associated with neurodegenerative diseases, such as Alzheimer's disease (AD). An scRNA-seq-based cellular atlas of the aging prefrontal cortex revealed heterogeneity in the aging patterns of different cell types in the human brain [18]. In particular, two distinct subpopulations of microglia, Mic.12 and Mic.13, which both express genes associated with susceptibility to AD. These subpopulations were linked to the accumulation of Aβ protein and the deposition of tau protein, respectively, ultimately diverging into different trajectories leading to AD or other types of brain aging.

In addition, scRNA-seq studies on sex differences provide evidence that the ovary and testis are critical organs reflecting divergent aging processes. For example, Isola et al. [19] revealed distinct mechanisms of ovarian decline in mice and humans. In mice, ovarian aging is primarily characterized by a decrease in follicle numbers, whereas in humans, it is associated with decreased oocyte quality and increased expression of cellular senescence markers such as CDKN1A and P21 [20]. Both species, however, exhibit increased immune cell infiltration during ovarian aging, particularly lymphocytes. Pyroptotic macrophages in the ovary reshape the immune microenvironment by producing molecules such as nitric oxide (NO) and reactive oxygen species (ROS), further accelerating the aging of stromal cells and reproductive decline.

Similarly, single-cell studies on testicular aging have revealed significant transcriptomic changes in Sertoli cells and spermatogonial stem cells, triggering inflammatory responses, oxidative stress, cellular senescence, and impaired DNA repair [21]. In addition, increased numbers of senescence-specific macrophages aggravate inflammation and fibrosis in testicular tissue by releasing cytokines and ROS, further affecting spermatogenesis. Notably, a senescence-specific macrophage (subtype 3) has been identified in mice but has not yet been observed in humans. This subtype is primarily involved in monocyte proliferation and T-cell activation and may be associated with an enhanced inflammatory response [22]. Lastly, testicular dysfunction in aging men has been shown to correlate closely with body mass index (BMI). These findings highlight how testicular aging involves complex molecular mechanism influenced by both aging and concurrent chronic diseases such as obesity.

The advancements in single-cell transcriptomics have not only transformed our understanding of cellular heterogeneity in aging but also redefined the scale at which we can interrogate aging mechanisms. By bridging the gap between molecular signatures and functional outcomes, scRNA-seq and related technologies provide an unparalleled opportunity to construct integrated, multi-dimensional models of aging. These models are poised to reveal the complex interplay of genetic, epigenetic, and environmental factors shaping the aging process. Moreover, the ability to map cellular trajectories and capture the emergence of senescence at single-cell resolution opens new avenues for identifying therapeutic targets and biomarkers. Such insights are critical for developing interventions to mitigate aging-related diseases, from neurodegenerative disorders like AD to reproductive decline [23]. Ultimately, single-cell transcriptomics stand as a cornerstone in the pursuit of precision geroscience, offering a roadmap to decipher the intricate molecular landscape of aging and laying the foundation for personalized approaches to promote healthy aging across diverse populations.

### Spatial omics

Spatial transcriptome technology enables the capture of spatial gene expression information in tissues by leveraging arrayed oligonucleotides, achieving resolutions comparable to the size of a single cell. In senescence research, this approach facilitates the identification of all cell-specific molecular markers and provides critical spatial information about senescent cells that is not available with scRNA-seq or snRNA-seq [24]. However, the limited sequencing depth of current spatial transcriptomics technologies and the low abundance of senescent cells in vivo pose significant challenges in mapping the spatial profiles of these. Therefore, integrating spatial omics with single-cell transcriptomics has become a dominant trend in current analyses.

Recent years have witnessed rapid advancements in spatial transcriptomics, with the development of technologies such as laser capture microdissection (LCM), in situ hybridization, MERFISH, Slide-Seq, HDST, DBiT-seq [25, 26]. These methods provide novel perspectives on understanding the spatial architecture and biological functions of complex tissues. For instance, Rodriques et al. [27] developed Slide-seq, which enables genome-wide expression analysis at a high spatial resolution of 10 μm by transferring RNA from tissue sections to DNA-barcoded beads and inferring the location of the RNA by sequencing. While Slide-seq marked a significant milestone in spatial transcriptomics, its limited sensitivity constrined its application to certain biological questions. To address this, Slide-seqV2 improved the technology by enhancing microsphere synthesis, library generation, and array sequencing steps, resulting in a tenfold increase in sensitivity [28]. By combining scRNA-seq with Slide-seqV2, SpatialScope allows for more accurately identifying senescence-related markers in different cell types and studying their spatiotemporal expression patterns in across tissues [29].

Spatial transcriptomics have been applied to map various organs and whole-body tissues in the spatial dimension. For example, spatial transcriptome maps of the mouse brains identified 27 distinct spatial regions and demonstrated that aging-associated gene expression changes are particularly pronounced in areas such as the isocortex, the hippocampal formation, brainstem and fiber tracts [30]. Moreover, aging alters inter-regional communication within the brain, including dysregulation of the WNT and collagen signaling pathways. Another study focused on specific brain regions, such as the frontal cortex and striatum, and used single-cell spatial transcriptomics to reveal distinct spatial distributions of cell types. For instance, vascular cells were found to cluster together, while microglia and astrocytes exhibited a more uniform distribution [31]. Futhermore, non-neuronal cells showed significant spatially dependent changes, for example, astrocyte activation in the corpus callosum of the aging brain appeared to be more spatially heterogeneous than that of microglia.

In human ovarian studies, spatial transcriptomics have revealed differences in the distribution of granulosa cells (GCs) in different regions of the ovary. GC subtypes 1 and 2 were predominantly present in young and middle-aged ovaries, whereas subtype 3, which is widely distributed in antral follicles, generally existed in elderly ovaries [32]. This shift suggested a decrease of functional GC types and the potential increase of apoptotic GC populations. On this basis, researchers constructed cellular maps of female reproductive organs in mice during reproductive cycle, pregnancy, and aging. These studies observed that immune cells were enriched in the upper reproductive tract, while the cervix and vagina were predominantly consisted of innate immune cells [33].

A recent breakthrough in spatial transcriptomics applied to multiorgan senescence revealed a spatial transcriptomic map of mammalian tissues and pinpointed Senescence-sensitive Spots (SSS) through a spatial senescence scoring system [34]. These regions exhibited disrupted tissue organization and loss of cellular identity, a phenomenon termed structural entropy increase. Additionally, the study introduced the concept of Immunoglobin-associated Senescence Phenotype (IASP), providing a novel theoretical framework for preventing and intervening in aging-related diseases.

Compared to single-cell transcriptomics alone, single-cell spatial transcriptomics provides a more comprehensive characterization of cell types, their spatial distribution, and inter-regional communications, enabling the identification of previously unrecognized cell subtypes and molecular markers. Through the analysis of spatial omics, the position and interrelationships of cells within tissues could be observed. This advanced technique is particularly instrumental in delving into and comprehending the complex aging mechanisms of organs such as the brain and paves the way for innovative strategies in the development of targeted interventions against aging and associated diseases.

### Single-cell epigenetics

Single-cell epigenetics investigates how epigenetic modifications regulate gene expression and cell phenotypes at the individual cell level, independent of changes in genomic sequences. The "epigenetic code" governing these processes includes DNA methylation, histone modifications, chromatin accessibility, and nucleosome positioning [35]. Dysregulation of these mechanisms has been implicated in aging acceleration. To explore these processes at high resolution, various single-cell sequencing techniques have been developed, leading to the generation of epigenetic heterogeneity maps across multiple tissues, organs, and cell types.

In 2015, researchers introduced techniques to profile chromatin accessibility and histone modification at the single-cell level, including single-cell DNase sequencing (scDNase-seq), single-cell Assay for Transposase-Accessible Chromatin using sequencing (scATAC-seq), and and single-cell chromatin immunoprecipitation sequencing (scChIP-seq) [36-38]. The continuous advancement of technology has overcome limitations in cell throughput seen in traditional bulk techniques while enhancing sequencing efficiency and data quality. For DNA methylation analysis, single-cell genome-wide bisulfite sequencing (scBS-seq) and single-cell methylome and transcriptome sequencing (scM&T-seq) are widely used [39, 40]. These methods utilize bisulfite treatment to convert unmethylated cytosine into uracil, followed by high-throughput sequencing to infer methylation patterns at single-nucleotide resolution.

For histone modifications, scChIP-seq and the cleavage under targets and tagmentation (CUT&Tag) method effectively characterize and histone modification landscapes [41]. Chromatin accessibility is typically assessed using scATAC-seq, which utilize Tn5 transposase to insert sequencing adapters into open chromatin regions, enabling the identification of regulatory elements at single-cell resolution [42]. Meanwhile, single-cell micrococcal nuclease sequencing (scMNase-seq) provides additional insights into nucleosome positioning and chromatin remodeling by selectively digesting unprotected linker DNA and sequencing the remaining nucleosome-associated fragments [43].

Additionally, three-dimensional chromatin structure can be explored using chromatin conformation capture (3C) and its derivatives, such as Hi-C (High-throughput chromosome conformation capture) and ChIA-PET (chromatin interaction analysis by paired-end tag sequencing), which utilize restriction endonucleases to fragment and re-ligate DNA to reveal chromatin interactions [44]. These single-cell epigenetic techniques have significantly advanced our understanding of gene regulatory networks and cellular heterogeneity in aging.

### Epigenetic Clocks and Aging

Epigenetic clocks estimate an individual's biological age based on epigenetic modifications, such as DNA methylation and histone modifications, often providing higher predictive accuracy than chronological age. Age-associated changes in DNA methylation occur at specific genomic loci, termed clock-like differential methylation loci (ClockDML). The EpiTrace algorithm uses scATAC-seq to quantify chromatin accessibility at these loci, allowing inference of cell division history and developmental [45]. Chromatin accessibility at ClockDML sites has been observed to decrease with successive cell divisions, a process independent of DNA methylation yet highly correlated with predicted biological age. This suggests that these epigenetic modifications may contribute to aging through interconnected biological pathways.

A statistical framework, scAge, predicts the cellular epigenetic age by calculating the probability between cell methylation status and age [46]. By tracking the aging process in liver cells, muscle stem cells, and embryonic stem cells, scAge revealed distinct epigenetic aging patterns. Notably, liver and muscle stem cells exhibited slower epigenetic aging, whereas embryonic stem cells underwent transient rejuvenation. Furthermore, epiblast-derived cells showed a natural reduction in epigenetic age during embryonic development, highlighting stratified rejuvenation processes in early life.

### Histone modifications and epigenetic aging in immune cells

The epigenetic landscape profiling using cytometry by time-of-flight (EpiTOF) has revealed that chromatin modification heterogeneity increases with age at both the single-cell and inter-individual levels [47]. Aging immune cells exhibit distinct histone modification patterns: most immune cells show increased levels of H3K27me2 and reduced levels of H3K27ac, whereas certain CD4+ and CD8+ T cells subsets display different histone modification patterns [48]. These findings highlight the dynamic epigenetic landscape in immune aging, suggesting that histone modifications may serve as biomarkers for immunosenescence.

To further investigate histone modifications in aging, researchers developed the ImAge algorithm, which quantifies the spatial distribution of histone modification through microscopic imaging and constructs cellular aging trajectories [49]. Analysis across multiple organs revealed that aging tissues exhibit elevated levels of H3K9me3, H3K27me3, and H3K27ac, leading to progressive chromatin heterochromatinization. Interestingly, this pattern contrasts with the previously described H3K27ac reduction in aging immune cells, suggesting that histone modifications follow tissue-specific aging trajectories influenced by distinct regulatory mechanisms.

Beyond natural aging, recent studies indicate that epigenetic reprogramming interventions can restore youthful chromatin states. Both caloric restriction and OSKM factors (OCT4, SOX2, KLF4, MYC)-induced reprogramming have been shown to partially reverse aging-associated histone modifications, supporting their potential role in epigenetic rejuvenation [50, 51].

These findings not only underscore the complexity of age-related DNA methylation and chromatin remodeling, but also further emphasize the central role of single-cell histone modification profiles in the in-depth understanding and potential mitigation of epigenetic aging. Particularly, the study of epigenetic aging clocks enables us to precisely depict the molecular trajectory of aging at the single-cell level. Meanwhile, the application of a series of cutting-edge single-cell epigenomic technologies has brought unprecedented resolution and depth to aging research, allowing researchers to deeply explore novel regulatory sites and mechanisms, thereby developing precise anti-aging strategies targeting specific cells or modifications.

### Single-cell metabolomics

Single-cell metabolomics provides a powerful approach to decipher metabolic heterogeneity at the cellular level, enabling the investigation of phenotypic changes and metabolic mechanisms across diverse cell populations. This field is particularly crucial for aging research, as metabolism plays a central role in cellular homeostasis, stress responses, and longevity-associated pathways.

Currently, single-cell metabolomics mainly relies on mass spectrometry (MS), nuclear magnetic resonance spectroscopy (NMR), and chromatographic techniques [52], which facilitate the real-time characterization of cellular biochemical states. However, due to the low abundance and rapid turnover of metabolites, achieving high-throughput and high-sensitivity detection at the single-cell level remains a significant challenge.

To address these limitations, various advanced techniques have been developed. For example, mass spectrometry-based methods, particularly matrix-assisted laser desorption/ionization mass spectrometry imaging (MALDI-MSI), have been widely employed in single-cell lipidomics and metabolic profiling. MALDI-MSI enables the visualization of metabolic heterogeneity across different cell types, providing insights into the spatial distribution of metabolites and their association with cell fate [53]. Another approach nanoelectrospray ionization-MS (nanoESI-MS), has been combined with single-cell patch clamp technology to achieve in-situ, real-time, and label-free analysis in living cells, facilitating the detection of a diverse range of metabolites.

In addition to traditional MS, spatially resolved single-cell metabolomics has emerged as a powerful strategy for integrating metabolite profiling with tissue architecture. A recent advancement in this area is the Single Cell Spatially resolved Metabolic (scSpaMet) framework, which combines Time-of-Flight Secondary Ion Mass Spectrometry (TOF-SIMS) with Imaging Mass Cytometry (IMC) to enable the simultaneous characterization of metabolite profile and protein markers in the same tissue section [54]. This approach provides a multi-dimensional view of cellular metabolism, linking metabolite dynamics to specific cell types and microvironmental interactions.

Another promising innovation in single-cell metabolomics is the integration of high-throughput microfluidic technologies. For instance, the SlipChip-SERS platform, which combines microfluidic SlipChip device with surface-enhanced Raman spectroscopy (SERS) technology, offers ultrasensitive, label-free, and high-throughput metabolic detection at the single-cell level [55]. By encapsulating individual cells within microdroplets and capturing the Raman signals of released metabolites using metal nanoparticles, such as spermine, which is associated with increased β-galactosidase activity and reduced LMNB1 level, both hallmark features of senescence [56]. The ability to link metabolic alterations to cellular aging processes highlights the potential of single-cell metabolomics in advancing our understanding of aging-related metabolic dysregulation.

Despite the significant advances in single-cell metabolomics, quantifying metabolite dynamics at the single-cell level remains highly challenging. The abundance of metabolites in a single cell is typically low and can fluctuate significantly due to intrinsic metabolic heterogeneity, dynamic cellular states, and micro-environmental influences [57]. These factors make it difficult to capture real-time metabolic fluxes and determine the temporal dynamics of metabolite changes within cells. Therefore, single-cell metabolomics is still in its developmental stage, particularly in the field of aging research. To circumvent these limitations, recent studies have increasingly focused on integrating metabolomics techniques with single-cell transcriptomics to reveal the associations between plasma metabolites and gene expression in different cell types, indirectly reflecting changes at the cellular level. This approach provides a more comprehensive view of cell-type-specific metabolic alterations that occur during aging.

A prime example of this integrative approach is the combination of non-targeted liquid chromatography-mass spectrometry (LC-MS) plasma metabolomics with scRNA-seq [58], which has revealed significant correlations between aging-associated plasma metabolites and gene expression modules in specific cell types, particularly in adipose and muscle tissues. Specifically, biosynthetic enzyme-encoding genes associated with tRNA modification-including N1-methylinosine, N2,N2-dimethylguanosine, and N6-carbamoylthreonine adenosine, exhibited age-related upregulation in these tissues. These findings suggest that age-dependent shifts in tRNA modifications may contribute to metabolic alterations during aging.

Furthermore, the integration of single-cell sequencing with NMR-based plasma metabolomics has led to the identification of a comprehensive panel of aging-related metabolite biomarkers spanning amino acids, ketone bodies, fatty acids, and lipoprotein lipids [59]. Among these, glycoprotein acetylation (GlycA) emerged as the most robustly correlated biomarker with the risk ratio for all-cause mortality, reinforcing its potential as a systemic aging marker. In addition, this study uncovered previously unrecognized metabolite biomarkers linked to lipoproteins, shedding light on the intricate connections between metabolic dysregulation and aging-associated pathophysiology.

### Single-cell proteomics

Single-cell proteomics, when combined with single-cell metabolomics, provides a comprehensive view of cellular states, focusing on the protein expression, post-translational modification (PTMs), and functional interactions [60]. Unlike transcriptomic approaches, proteomics directly measures the functional molecular effectors of cellular processes, making it particularly relevant for studying aging-related phenotypic changes. MS-based single-cell proteomics has been instrumental in uncovering age-associated proteomic alterations. For instance, a comparative analysis of mouse and human oocytes found that in aging mouse oocytes, the translation efficiency of most m6A-modified genes is significantly reduced, a process mainly mediated by YTHDF3 and its key target gene Hells [61]. However, in human oocytes, RNA translation efficiency appeared independent of m6A modification, and instead, RNA binding proteins (RBPs) exhibited significantly differential expression during senescence, suggesting a distinct mechanism of translational regulation in humans

Despite its potential, the core challenge of single-cell proteomics lies in the limited quality of proteins within individual cells, making it particularly difficult to capture low-abundance proteins using conventional MS techniques [62]. Early studies primarily relied on labeling-based techniques, such as flow cytometry and immunofluorescence, which utilize fluorescent dyes or antibodies to detect specific proteins. However, these methods have inherent limitations, including cell structure disruption due to fixation and permeabilization steps, as well as a restricted capacity to measure a broad range of proteins simultaneously [63].

Recent advances in MS-based single-cell proteomics have significantly improved the sensitivity and depth of protein quantification. Notably, Minimal ProteOmic sample Preparation (mPOP) and tandem MS tags (TMT) labeling-based SCoPE2 have optimized the experimental pipeline for single-cell proteomics [64]. The ScoPE2 system uses TMT to label proteins from carrier samples, reference samples, and single cell lysates, followed by peptide fragmentation and quantitative MS detection. The multiplexing capability of TMT labeling allows for high-throughput and accurate protein quantification, overcoming prior limitations in sensitivity and reproducibility.

In addition to MS-based techniques, cytometry by time-of-flight (CyTOF) has emerged as a powerful approach for single-cell proteomics. By integrating MS and flow cytometry principles, CyTOF overcomes the limitation of overlapping fluorescence spectra of flow cytometry, thereby enabling multiplexed protein detection across diverse immune cell populations. The immune systems of aging mice and humans were analyzed by CyTOF and single-cell protein analysis, which can detect the differences in the expression of protein markers of different immune cell subsets, further clarify the characteristics of these subsets and the phenotypic remodeling and functional changes during aging [65]. Specifically, the study identified elevated Fn1 expression in fibroblasts, which was related to pro-inflammatory SASP factors, including IL6, CCL2, and CXCL1, highlighting its potential role in age-related inflammatory processes.

Altogether, aging-related metabolites and protein translation modifications in single cells have a crucial impact on the function, localization and interaction of protein. With high sensitivity analysis technology, single-cell proteomics can accurately identify the abnormal modifications of specific proteins and intervene in a timely manner in the physiological processes that accelerate aging, such as cell cycle arrest and metabolic alterations. This holds the promise of enabling a deeper comprehension of the essence of cell aging and offers the potential to unearth novel therapeutic targets.

**Table 2:** Application of machine learning in single cell aging research.

Study	Dataset	Model	Structure	Application
**Zhang et al. [59]**	UK Biobank category 220	LASSO	Linear	Metabonomics aging score
**Zhu et al. [71]**	GSE213516	PLSR	Linear	Single-cell aging clock
**Mao et al. [72]**	Tabula Muris Senis	EN	Linear	Single-cell biological age model
**Olinger et al. [73]**	Baltimore Longitudinal Study of Aging (BLSA)	EN	Linear	Identifying senescence-associated biomarkers
**Tao et al. [74]**	52 senescence-related studies	SVM-RFE	Linear	Classification of senescent cells into six SIDs
**Wu et al. [76]**	GEO EGA Array Express	CT	Tree	EC.SENESCENCE.SIG
**Duran et al. [78]**	Human cell lines (A549, SK-HEP-1, SK-MEL-103, MCF7, HCT116, IMR90) and mouse liver tissue samples	CT RF	Tree	Cellular senescence score and tissue senescence score
**Hajdarovic et al. [81]**	GSE188646	XGBoost	Tree	Predicting neuron age and identifying aging markers
**Kusumoto et al. [84]**	DRA010959	CNN	Deep learning	Deep-SeSMo senescence scoring system
**Qu et al. [85]**	WI-38, HCA2, IMR-90, HUVEC et al.	DeepScence	Deep learning	Automatic encoder based on CoreScence genes

### Identifying senescence markers at the single-cell level: leveraging machine learning algorithms for precision aging biology

**Figure 2.**
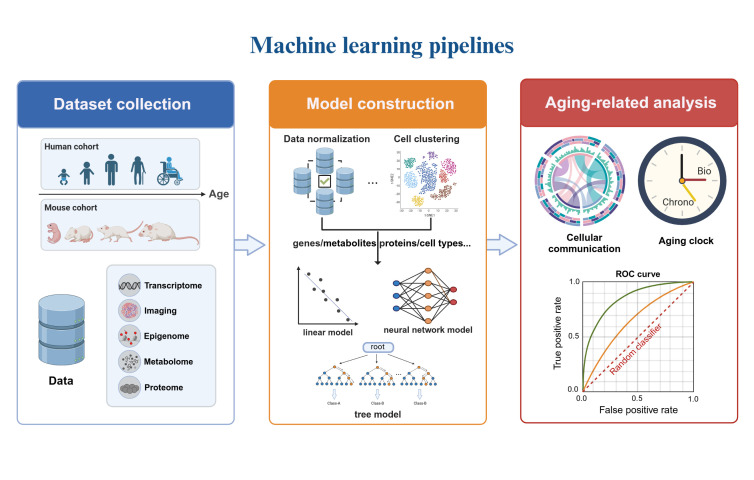
**AI-enhanced pipelines for unraveling single-cell aging mechanisms**. The flowchart describes the process of analyzing single cell aging using machine learning models. It starts with data collection and preprocessing of single cell queue. Through the steps of model construction-feature selection-aging related analysis, the biological mechanisms of aging are revealed. Figure created using BioRender.

Machine learning models can be broadly classified into four categories based on their structure: linear models, tree-based models, graph models and neural networks [66]. Each model type is suited to specific data types and analysis tasks, and selecting the appropriate parameters and training strategies is crucial for optimizing model performance. In this study, we focus on algorithms based on linear models, tree-based models, and neural networks to identify cell senescence markers at the single-cell level (Table 2). Usually, the standardized genes or protein features are input into the feature selection model, to identify crucial factors related to aging. Based on optimal markers, the models are then employed to classify phenotypes or construct aging clocks, and other comprehensive analyses (Fig. 2). In summary, this field strives to elucidate the intricate biological mechanisms that underline the complex aging phenomenon, thereby contributing to a deeper understanding of its molecular and cellular basis.

### Identification of senescence markers based on linear models

Linear models are statistical models that assume a linear relationship between input features and outputs, and are widely used in machine learning [67]. Among these, linear regression and logistic regression are the most common approaches, used for regression and classification tasks, respectively. In single-cell aging research, linear models identify biomarkers related to aging by analyzing changes in gene expression across different cell populations during aging. For instance, linear regression can be employed to identify genes with significant expression changes over time, helping to infer their role in the aging process. Logistic regression, on the other hand, is commonly used for binary classification. By transferring the linear combination of input features to a Sigmoid function, it outputs a probability value between 0 and 1, which is used to distinguish aging cells from non-aging cells, thus enabling accurate prediction of the aging state.

### Least absolute shrinkage and selection operator regression

Feature selection and dimensionality reduction are essential steps when processing high-dimensional single-cell data [68]. Common regularization methods such as least absolute shrinkage and selection operator (LASSO) and Ridge regression are used to identify key features related to aging while mitigating the risk of overfitting through L1 and L2 regularization (penalty term), respectively. LASSO regression, in particular, is valuable for identifying aging markers, as aging-related genes often exhibit low expression levels. By applying LASSO, the model compresses less influential feature coefficients to zero, effectively highlighting genes most strongly associated with aging and excluding irrelevant or redundant features.

Senescence scores are a crucial metric for quantifying the aging process and have widely gained usage in biomedical research. Using the LASSO method, one study identified metabolic markers related to aging from 325 NMR biomarkers in UK Biobank database [59]. This study further explored the causal relationship between these metabolic markers and aging-related diseases through multivariable Mendelian randomization (MVMR) analysis. Additionally, the researchers constructed a metabolomics-based senescence score model, which correlated with established aging indicators such as MetaboHealth and leukocyte telomere length [69, 70]. This model was shown to effectively reflect individual aging status and demonstrated strong performance in predicting short-term mortality risk and identifying individuals with accelerated aging.

### Partial least square regression

Biological age provides a more accurate measure of aging than chronological age, as it reflects the rate of aging associated with functional decline. Researchers have developed a senescence clock model to predict the biological age of supercentenarians (SCs) [71], which utilizes partial least square regression (PLSR) to analyze the proportions of age-related cell types in peripheral blood mononuclear cells (PBMCs). This model calculates a senescence score that reflects the cellular state and shows a strong correlation with the chronological age of the individual’s cells. The study revealed distinctive senescence delay characteristics in both cell type proportions and gene expression in SCs. For instance, the decline of CD8+ Naive T cells and Naive B cells was delayed in SCs, as was the downregulation of ribosome- and translation-related genes in cell types with high ribosomal activity.

### Elastic network regression

Elastic net (EN) regression combines the strengths of both LASSO and ridge regression, making it well-suited for situations where the number of features exceeds the number of samples. To quantify cellular senescence across different tissues, Mao et al. [72] developed a tissue-specific single-cell biological age model called the Single-Cell Aging Level Estimator (SCALE). This model employs a guided forward selection strategy combined with EN to construct a unique SCALE model for each tissue type. When compared to the scAge single-cell aging clock using epigenetic markers, SCALE outperformed in capturing biological age, reflecting senescence differences of muscle stem cells, and predicting previously unknown cell types. The model also identified new potential senescence-related genes, such as Lars2 and Rpl13a, whose expression changes were related to aging-related diseases and physiological function alterations. Interestingly, the study revealed a complex trend in single-cell entropy, showing increasing but variable changes during aging. Similarly, Olinger et al. [73] applied elastic net modeling to analyze senescence- SASPs secreted by senescent monocytes, successfully identified a set of protein markers, which were defined as elastic net-selected proteins (ENSPs). These 21 markers can accurately predict various clinical features closely related to aging and obesity, including inflammation, HDL levels, BMI, and waist circumference. In addition, PCA analysis mapped the expression levels of high-impact panels to a single continuous variable, representing the aging process in a more unified manner.

### Support vector machine

Support vector machine (SVM) differs from traditional linear regression methods by distinguishing between different classes of samples using an optimal hyperplane, which is mainly applied to classification tasks. More importantly, SVM can efficiently handle nonlinear problems by utilizing the kernel trick, which maps data from the original to a higher-dimensional feature space where the problem may become linearly separable. For example, using a radial basis function (RBF) kernel, SVM can map data to a potentially infinite-dimensional space, enabling the model to handle complex, nonlinear decision boundaries. Here, we will focus on linear SVM.

Tao et al. [74] developed a machine learning program called SenCID. The SVM model achieves an average AUC value of over 0.9 in distinguishing senescent cells from non-senescent cells, outperforming a variety of machine learning methods and Gene Set Variation Analysis (GSVA) methods. When combined with recursive feature elimination (RFE) method, the model accurately classifies different types of cells into six major senescence IDs (SIDs), thereby categorizing cellular senescence status with precision. The research findings revealed significant differences in senescence characteristics across distinct SIDs. Notably, signaling entropy exhibited a decreasing trend with age, which contrasts with the concept of increasing entropy in biological systems from a thermodynamic perspective [75]. In biological systems, entropy should be more precisely integrated with biological functions and information processing mechanisms. The reduction in signaling entropy is mainly due to the decrease of the ability of organisms to maintain complex signal networks, which leads to the decrease of the functionality and complexity of signal transmission systems.

## Identification of senescence markers based on tree models

### Decision tree

Tree-based machine learning models are broadly classified into decision tree and ensemble tree model, with the latter category including techniques like random forest (RF) and gradient boosting decision tree (GBDT). Decision tree can further be divided into classification tree (CT) and regression tree (RT), depending on the nature of the task-classification or regression. In biological research related to aging, the decision trees partition the sample space based on specific biological features or conditions. For instance, when gene expression levels from scRNA-seq data are used as features, each internal node in the tree represents a test on a feature, and the leaf nodes indicate either class labels (for classification tasks) or numerical predictions (for regression tasks). Decision trees are particularly useful for constructing decision paths based on the expression levels of genes, thereby enabling the classification of cells based on phenotypic traits associated with aging. These decision paths can help to identify genes or gene expression patterns that are indicative of aging-related cellular changes.

Decision trees are typically combined through weighted aggregation to form strong classifiers, demonstrating superior classification performance compared to individual decision trees. Specifically, Wu et al. [76] employed a model that aggregated multiple classification trees to identify key genes influencing the immune responses and therapy outcomes in cancer patients. By leveraging the AdaBoost classification tree method, they developed the senescence-related transcriptomic signature (EC.SENESCENCE.SIG), which provided a more comprehensive predictive framework than single genetic marker, such as tumor mutational burden. Their findings revealed that the senescence level of tumor endothelial cells (TEC) was significantly higher than that of tumor cells and other vascular related cells, highlighting the potential role of endothelial cell senescence in tumor progression and therapeutic resistance.

### Random forest

Random forest is a machine learning method based on an ensemble of decision trees, which excels at integrating a wide range of senescence-related features, particularly when dealing with complex single-cell multi-omics data. It constructs multiple decision trees using a large number of single-cell samples. Each tree is built from bootstrap samples derived from the original training dataset [77], with the node segmentation process repeated until a predefined stopping condition is met. The predictions from all decision trees are aggregated via majority voting for classification tasks, or averaging for regression tasks, to determine the senescence state of cells or classify them into specific senescence-related subgroups. In addition, RF provides variable importance scores for each feature, allowing researchers to identify key factors associated with aging.

A single decision tree often fails to capture the complexity of research outcomes, which is why it is commonly used in conjunction with random forests. The CT-RF model represents an innovative computational framework that skillfully integrates three algorithms [78]: classification tree construction, random forest probability estimation, and the voting consensus algorithm. By using the A549 etoposide model (AEM) and A549 etoposide random forest model (AERFM) sub-models to analyze microscopic characteristics of mononuclear cells- such as nuclear area and radius of gyration-this framework can accurately evaluate the aging state of cells.

These models showcase their distinctive capabilities in several critical areas: they not only differentiate senescent cells from quiescent ones with high precision, but also remain unaffected by variations in cell density. Furthermore, they effectively capture the intricate associations between nuclear features of cells and DNA damage. The predictive precision of these classifiers has been robustly validated through extensive experimental testing across diverse cell types, with outcomes that align closely with standard senescence detection methods, such as SA-β-Gal staining.

Moreover, the application of the model has been extended through the incorporation of the cellular senescence score, which subsequently facilitated the development of the tissue senescence score (TSS). When combined with senescence markers such as p21 and p16INK4a, the TSS not only quantifies the level of senescence within tissues but also predicts cellular alterations linked to liver fibrosis and aging in mice. The organic integration of these three algorithms enhances the model's stability and predictive power, equipping researchers with a potent and dependable tool for the identification and quantification of cellular senescence.

### GBDT

GBDT is based on the boosting ensemble learning approach, where a series of decision trees are trained sequentially [79]. In Each iteration, the model focuses on the residual errors between the previous predictions and the true aging states, gradually improving its ability to distinguish gene expression patterns characteristic of senescent cells. The final prediction is obtained by aggregating the outputs of all decision trees. XGBoost, an enhancement of GBDT [80], introduces a regularization term in its objective function to mitigate over-fitting, alongside a more efficient approximation algorithm that enables it to handle large-scale datasets effectively. This makes XGBoost particularly advantageous in single-cell research. For example, XGBoost can integrate diverse data types-such as gene expression, protein abundance, and epigenetic modification-allowing for precise predictions of cellular aging states and types. Moreover, it can identify key features within the model, providing valuable insights into the complex molecular mechanisms and cellular heterogeneity of aging at the single-cell level. In a prospective study, researchers investigated single-cell characterization of the hypothalamus in female mice [81]. Using advanced snRNA-seq technology, they analyzed the hypothalamus of both young and aged female mice. A supervised learning model was constructed, utilizing neuronal subtypes as key features for classification. The XGBoost classifier outperformed other models, achieving an accuracy of 78%. Subsequently, the researchers fine-tuned the model by optimizing hyperparameters and retraining it several times to further enhance its predictive performance. An analysis of feature importance revealed that the expression of Xist, a long non-coding RNA involved in X-chromosome inactivation, as well as specific genes such as Ftx, Jpx, and Tsix, were significantly upregulated in the hypothalamus of female mice. Notably, these expression changes strongly correlated with neuronal aging. Of particular interest is the finding that this gene signature could serve as a distinct marker of brain region specificity in female patients with AD. However, these features were not detected in adult male mice. The high accuracy of the XGBoost model in predicting neuronal age provides new insights into the mechanisms underlying organ and cellular aging, offering potential avenues for future research into age-related neurodegenerative disease.

### Identification of senescence markers based on neural network model

Neural network models typically composed of input layers, hidden layers, and an output layer. Each neuron receives input signals from the preceding layer [67], processes these signals through weighted summation and activation functions, and then passes the output signals to the next layer, enabling the network to learn and distinguish complex patterns within the data. Neural networks excel at automatically identifying intricate patterns and feature representations in raw data, leveraging their strong nonlinear fitting ability [82]. Common neural network types include multilayer perceptrons, convolutional neural networks (CNNs), and autoencoders.

Among these, CNNs are particularly well-suited for processing grid-like data, such as images, by automatically extracting relevant features through convolutional, pooling, and fully connected layers [83]. Kusumoto et al. [84] used CNN to build a deep-learning-based senescence scoring system named Deep-SeSMo, The model was trained using image data from human umbilical vein endothelial cells (HUVECs) subjected to various stressors, aiming to identify senescent cells by analyzing both peripheral and internal image features. The results demonstrated that the model achieved a high accuracy of 0.93 in distinguishing senescent cells from control cells. In addition, the senescence score, derived from the average output probability of the CNN model, exhibited a strong linear correlation with the intensity of the strssors. Furthermore, the study validated the anti-senescence and anti-aging properties of terreic acid, noting its ability to maintain mitochondrial function under stressful conditions, thereby further confirming the potential therapeutic value of the compound for age-related diseases.

Large-scale datasets are often affected by various technical noises during acquisition and processing, which can hinder the accurate analysis of the true cellular. To address this challenge, a deep learning-based algorithm called DeepScence has been developed [85]. This algorithm is designed to capture key features and potential patterns within gene expression data. Based on a custom zero-inflated negative binomial autoencoder, DeepScence offers advantages such as data imputation, allowing it to handle sparse and incomplete data more effectively. It utilizes a core senescence gene set, CoreScence, to capture senescence-related information, demonstrating superior performance in terms of recognition accuracy and F1 score.

**Figure 3.**
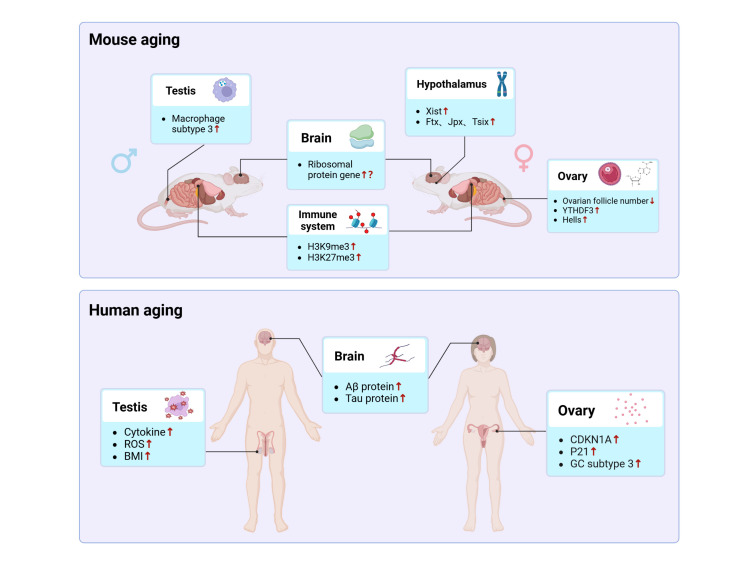
**Interpreting mouse and human aging at single-cell resolution**. Figure created using BioRender. Abbreviations: Xist, X-inactive specific transcript; YTHDF3, YTH domain family 3; Hells, Helicase, lymphoid specific; ROS, Reactive Oxygen Species; BMI, Body Mass Index; CDKN1A, Cyclin Dependent Kinase Inhibitor 1A; GC, Granule Cells.

In summary, through comprehensive analyses conducted on both young and old mice as well as human subjects, researchers have explored the aging mechanism in various organs, including the brain, ovary, and other organs. They deciphered novel mechanisms of cellular aging from multiple aspects, including gene expression regulation, histone modifications, and metabolite alterations (Fig. 3). A range of new senescence biomarkers were identified, offering new insights for understanding the complexity of cellular aging.

### Challenges and future directions in single-cell omics for aging research

The rapid advancements in single-cell multi-omics have provided unprecedented resolution for aging research, enabling researchers to dissect the molecular and functional heterogeneity of senescent cells. Despite significant progress, several challenges remain, including issues in data integration, computational interpretability, biomarker identification, and clinical translation. Here, we discuss these key challenges and propose future directions for advancing single-cell aging research.

### Challenges and future directions in single-cell multi-omics data integration

Aging is a highly complex process involving dynamic changes at multiple molecular levels, including gene expression, metabolism, epigenetic modifications, and protein interactions. However, integrating these distinct omics layers remains a significant challenge due to their differences in temporal resolution and biological context.

For example, transcriptional changes may occur from minutes to hours, whereas epigenetic modifications and proteomic alterations may take days or longer to manifest. These differences complicate the development of unified models that accurately capture the multi-dimensional landscape of aging.

Furthermore, the inherent sparsity and noise in single-cell datasets make data integration even more challenging, potentially leading to information loss or misinterpretation. To address this, a variety of computational frameworks are dedicated to bridging the gap of data integration of single-cell multi-omics. Cao and Gao [86] proposed the graph-linked unified embedding (GLUE) computational framework, which explicitly models cross-omics interactions. By integrating variational autoencoders (VAE) with graph-guided embeddings, GLUE establishes feature space link, enabling the effective integration of gene expression, chromatin accessibility, and DNA methylation data from mouse cortical neurons. This approach has provided valuable insights into the contributions of different epigenetic regulatory mechanisms to gene expression.

Similarly, Cao et al. [87] developed uniPort, a data integration method that integrates the coupled-VAE and minibatch unbalanced optimal transport (Minibatch-UOT). It projects the highly variable common gene sets of different datasets into the potential space and reconstructs the data through a special decoder to deal with the heterogeneity between datasets. This method makes a substantial contribution to identifying mouse brain structure, the tertiary lymphatic structure of breast cancer and pancreatic cancer heterogeneity. In addition, a deep probabilistic framework MIDAS (mosaic integration and knowledge transfer) leverages VAE and self-supervised learning to align different modalities. This method successfully constructs a single-cell trimodal map of human peripheral blood mononuclear cells, enabling cell differentiation trajectory analysis and cross-tissue knowledge transfer, thereby improving data integration accuracy [88]. However, these methods still require further optimization, particularly in their ability to handle heterogeneous aging-related datasets. Future research should focus on developing aging-specific data fusion frameworks that can systematically incorporate multi-omics information while preserving cell-type-specific aging signatures.

Additionally, large-scale, cross-species, and cross-tissue reference datasets for single-cell aging research are still lacking. Although databases such as Aging Atlas [89] and Immunosenescence Inventory [90] have been established, they remain limited in standardization, making cross-study comparisons difficult. Establishing standardized protocols for single-cell aging data collection, processing, and annotation is essential for ensuring data reproducibility and facilitating meta-analyses across different studies.

### Improving the interpretability of computational models in single-cell aging research

With the increasing application of AI and machine learning in single-cell aging research, concerns regarding model interpretability have emerged. Deep learning models, while powerful, often function as “black boxes,” making it difficult to extract biological insights from their predictions. This lack of interpretability hinders the clinical applicability of AI-driven aging biomarker discovery.

To address this issue, explainable AI (XAI) approaches, such as Shapley Additive Explanations (SHAP) and attention-based neural networks, have been proposed to highlight key genes, metabolites, and proteins associated with aging [91]. Additionally, transfer learning, which allows models to leverage pre-existing biological knowledge, has shown promise in improving model generalizability while reducing the need for large training datasets [92].

Another key limitation is the assumption that aging biomarkers exhibit a linear relationship with chronological age. However, emerging evidence suggests that aging is a non-linear process, with certain metabolic pathways undergoing abrupt shifts at critical age milestones—referred to as "sudden aging" events [93]. Thus, future research should focus on developing deep learning frameworks that can capture these non-linear aging dynamics and integrate network-based approaches to reveal complex regulatory interactions [94].

### Discovery and clinical translation of aging biomarkers

The identification of robust aging biomarkers is critical for developing targeted interventions against age-related diseases. However, existing biomarkers are often derived from bulk tissue analysis, which fails to account for cellular heterogeneity. Moreover, the applicability of commonly used markers, such as p16INK4a, varies across cell types, limiting their utility in defining universal senescence signatures.

Single-cell omics have facilitated the discovery of novel senescence markers by enabling researchers to profile individual cells within complex tissues. For example, the integration of scRNA-seq and spatial transcriptomics has allowed for the construction of aging cell atlases at tissue-wide resolution, revealing cell-type-specific aging trajectories. Additionally, combining single-cell senescence biomarkers with large-scale human cohort studies (e.g., UK Biobank) has the potential to improve biological age estimation and advance personalized aging diagnostics.

A particularly promising area is the development of single-cell aging clocks, which extend beyond traditional methylation-based epigenetic clocks to capture cellular-level aging heterogeneity. Unlike existing aging clocks that estimate biological age at the tissue level, single-cell approaches can identify aging patterns in specific cell populations, paving the way for more precise age-related disease risk assessments.

### Application of single-cell technologies in anti-aging interventions

Beyond biomarker discovery, single-cell omics is increasingly being applied to evaluate the efficacy of anti-aging interventions. One of the most promising approaches is the targeted clearance of senescent cells using senolytics, which has been shown to improve tissue function and lifespan in preclinical models. However, the heterogeneous nature of senescent cells poses a major challenge, as different tissues may require distinct senolytic strategies.

Single-cell multi-omics profiling has been instrumental in identifying senescence-specific vulnerabilities that could serve as drug targets. For example, recent studies have combined single-cell transcriptomics with metabolomics to uncover metabolic dependencies in senescent cells, providing potential targets for therapeutic intervention. Moreover, single-cell proteomics have revealed how SASP factors vary across cell types, enabling the development of more precise senescence-modulating therapies.

Another exciting avenue is the use of partial reprogramming to reverse epigenetic aging. Studies have demonstrated that transient induction of Yamanaka factors can rejuvenate aged cells without inducing pluripotency. However, concerns remain regarding the long-term safety of reprogramming-based interventions. Single-cell sequencing can help address this by tracking epigenetic changes at high resolution, allowing researchers to fine-tune reprogramming protocols for safer applications in regenerative medicine.

### Future perspectives

The continued advancement of single-cell aging research will require a multidisciplinary approach, integrating cutting-edge computational methods with high-throughput experimental techniques. A key priority should be the development of AI-driven, multi-modal data integration frameworks that can simultaneously analyze transcriptomic, epigenomic, proteomic, and metabolomic data at the single-cell level. Additionally, global collaboration is needed to establish standardized, large-scale single-cell aging datasets that enhance data reproducibility and facilitate cross-study comparisons.

On the computational front, further improvements in biological interpretability are essential. Future research should focus on incorporating knowledge-based constraints into deep learning models, improving their ability to extract meaningful biological insights. Moreover, the development of non-linear aging models will be crucial for accurately capturing the complexity of aging trajectories.

In conclusion, while significant challenges remain, the field of single-cell aging research holds immense promise. As computational tools and experimental techniques continue to evolve, single-cell multi-omics is poised to revolutionize our understanding of aging, drive the discovery of new therapeutic targets, and ultimately contribute to strategies for healthy longevity and disease prevention.

## Discussion

The exponential advancement of machine learning technology has furnished powerful tools for biomarker prediction, significantly augmenting the frontiers of aging research. Nevertheless, the extent to which the predicted biomarkers authentically mirror the aging process necessitates corroboration through experimental validation and practical application. This is not only pivotal for establishing their scientific merit of aging research but also decisive in defining their prospective utility in clinical translation.

Among the metabolic biomarkers identified in the UK Biobank, GlycA exhibits the highest risk ratio (HR) in relation to all-cause mortality. Indeed, a 10-year follow-up study has revealed that GlycA serving as a crucial risk indicator for cardiovascular diseases, is closely associated with the decline in cardiovascular function commonly observed during the aging process [95]. An elevation in GlycA levels is frequently concurrent with the occurrence and progression of chronic inflammation within the body. This state can detrimentally affect vascular endothelial function, facilitate the development of atherosclerosis, and subsequently heighten the risk of thrombosis.

Secondly, Lars2 and Rpl13a emerge as novel potential genes associated with aging. Experimental evidence indicates that when Lars2 is knocked out in the hippocampus of mice, it results in an elevation of the ROS level, disrupts mitochondrial function, ultimately leads to cognitive impairment [96]. Conversely, the over-expression of Lars2 can mitigate neurodegenerative processes and reduce the accumulation of p-tau. The alteration in Lars2 expression is intricately linked to the progression of aging - related diseases, such as AD, and holds promise as a potential target for related therapeutic interventions. Similarly, the increasing level in Rpl13a snoRNAs upregulates the expression of Nox1, which in turn promotes the production of ROS [97]. The elevated ROS levels cause changes in the phospholipids of the erythrocyte membrane, facilitating the exposure of phosphatidylserine (PS), which provides binding sites for the deposition of complement C3 and C3a, thereby exacerbating the risk of thrombosis. In vivo experiments have also demonstrated that the length of the thrombus in elderly mice with Rpl13a snoRNA knockout is significantly shorter compared to the control group.

And as is well known, X chromosome inactivation serves as a fundamental mechanism for dosage compensation between mammalian females and males. Notably, its regulatory dynamics have been implicated in the pathogenesis of Parkinson’s disease (PD), a neurodegenerative disorder intricately linked to the aging process [98]. Studies have demonstrated that XIST facilitates PD progression by upregulating Sp1 and LRRK2 expression, this upregulation promotes apoptosis while suppressing cell proliferation, ultimately exacerbating the neurodegenerative process. MPTP-induced neurotoxicity resulted in severe brain damage in mice. However, it significantly alleviated neuronal injury when lentiviral-mediated shXIST knockdown was applied. Additionally, the expression of Ftx, Jpx, and Tsix genes plays a pivotal role in the initiation of X chromosome inactivation, exhibiting synergistic or compensatory interactions with Xist RNA transcription [99].

In the realm of aging biology research, some biomarkers predicted by diverse algorithmic models have been validated to a certain extent, laying the foundation for a more profound comprehension of the underlying aging mechanisms. Nevertheless, the existing research is not without its limitations. Specifically, the specificity and accuracy of some biomarkers remain areas that require further in-depth exploration. For example, the prediction by the DeepScence algorithm that terreic acid possesses anti-aging properties. While this prediction offers a novel direction for anti-aging research, regrettably, there is currently a lack of relevant literature that can offer robust support. At present, the majority of studies on terreic acid concentrate on its antibacterial properties, thus creating a research void between its predicted anti-aging characteristics and the existing research body [100]. This disparity can be attributed to multiple factors. On one hand, the prediction outcomes of different research models and algorithms may be affected by a multitude of elements, including data quality and quantity, model assumptions, and parameter settings. These factors can potentially introduce biases when predicting the functions of specific biomarkers. For instance, insufficient or inaccurate data can lead to flawed predictions, and inappropriate model assumptions may oversimplify the complex biological processes involved. On the other hand, aging is an extremely intricate research domain encompassing numerous interwoven physiological and pathological processes. A single algorithmic prediction often struggles to comprehensively and precisely mirror the role of biomarkers within the complex in-vivo environment.

To tackle these problems, future research can be approached from multiple angles. For instance, at the cellular level, terreic acid impacts the integrity of bacterial cell membranes during its antibacterial action. Interestingly, the cell membranes of aging cells also experience changes, thus it's worth delving deeper into whether terreic acid could influence cell aging by modulating membrane-related physiological processes. Regarding experimental verification, we need to comprehensively explore how various biomarkers impact aging-related indicators, including oxidative stress levels, inflammatory signaling pathways, and other key aspects. Furthermore, through the integration of more diverse biological information, we can establish a more robust and efficient approach to screen and validate potential anti-aging biomarkers, providing a solid scientific basis for future research on aging mechanisms and their clinical applications.
